# Self-Powered Piezoelectric-Biosensing Textiles for the Physiological Monitoring and Time-Motion Analysis of Individual Sports

**DOI:** 10.3390/s19153310

**Published:** 2019-07-27

**Authors:** Yupeng Mao, Mailun Shen, Bing Liu, Lili Xing, Song Chen, Xinyu Xue

**Affiliations:** 1 Physical Education Department, Northeastern University, Shenyang 110819, China; 2 School of Physics, University of Electronic Science and Technology of China, Chengdu 610054, China; 3 School of Arts, Beijing Sport University, Beijing 100084, China

**Keywords:** piezoelectric effect, biosensing, sports physiological monitoring, time-motion analysis, ZnO nanowires

## Abstract

Self-powered piezoelectric-biosensing textiles for the physiological monitoring and time-motion analysis of individual sports have been developed. The material system is composed of tetrapod-shaped ZnO nanowires on common textiles. The mechanism is based on the coupling of enzymatic reaction (LOx and lactate) and piezoelectric effect. After conformably attaching the device to the athlete, the device can monitor in real-time the moving speed, frequency, joint angle, and sweat lactate concentration of the athlete. The whole monitoring/analysis process is battery-free. The motor skills and physiological state of two athletes are investigated using the textiles, and different lactate threshold times and maximum lactate release capacities have been obtained. This technique can help them develop distinct training programs. This research is a new direction for the scientific monitoring of kinematics and may also stimulate the development of self-powered wearable sports-related systems.

## 1. Introduction

Individual physiological monitoring and time-motion analyses are important ways to improve performance in competitive sports [1,2,3,4,5,6,7,8,9,10,11,12,13,14,15,16,17,18,19]. Traditional technology usually requires a variety of devices working together for motion assessment. For example, video camera devices are usually used to capture exercise movement; GPS devices are used to record the running speed and real-time position [20,21,22,23,24], and various medical measurement devices are used to test blood lactate, heart rate, and other physiological parameters. These collected data are analyzed to evaluate the sports performance of individual athletes [3,6,13,17]. However, these devices have encountered some technical problems. Firstly, the devices are integrated together, and the system is usually in large size and cannot meet the demand of wearing on the body for real-time monitoring and analysis. Secondly, the body movement or position can be judged from the video or GPS data, but the tiny changes in joints or muscles under the skin cannot be estimated. Thirdly, the batteries or other power units embedded in the system also restrain the long-term and real-time utility due to the inconvenient recharge and replacement. Therefore, a new wearable battery-free sensing system for the real-time physiological monitoring and time-motion analysis of individual sports could have significant application in sports science. 

The emerging technology of self-powered flexible piezo-biosensors based on ZnO nanowires can enlighten our experimental design due to their distinct piezoelectric effect and excellent biocompatibility [25,26,27,28,29,30,31]. ZnO nanowires can harvest the mechanical energy (upon applied deformation) and output voltage/current signal through the piezoelectric effect. After the surface modification of enzyme or antibody on ZnO nanowires, the piezoelectric output is dependent on surface biochemical reactions and can be treated as the biosensing signal. The device can be driven by the body motion itself, realizing a battery-free system. A flexible polymer has also been introduced into the device, forming a wearable sensing system like an artificial electronic skin. Based on these previous works [32,33,34,35], we propose that this technique can be used for the physiological monitoring and time-motion analysis of individual sports. 

In this paper, self-powered piezoelectric-biosensing textiles for the physiological monitoring and time-motion analysis of individual sports have been developed. Tetrapod-shaped ZnO nanowires are located on the common textiles as the core structure of the device, and the textiles can be conformably worn on the athlete’s body like sportswear. The device can monitor the moving speed, frequency, and joint angle of the athlete in real-time (driven by the body motion itself). And the nanowires modified with lactate oxidase have the function of analyzing the sweat lactate concentration in real-time. The whole monitoring/analysis process is battery-free. The motor skills and physiological state of two athletes are investigated using the textiles, and different lactate threshold times and maximum lactate release capacities have been obtained. This multidisciplinary research opens up new directions for the scientific monitoring of kinematics and may promote the development of self-powered wearable sports-related systems.

## 2. Materials and Methods

### 2.1. Synthesis of ZnO Nanowires on Textiles

Tetrapod-shaped ZnO nanowires (T-ZnO, purchased from Chengdu Tianyou Jingchuang Technology, Chengdu, China) were firmly anchored on the textiles (many woven fabrics) using a PVDF (poly(vinylidene fluoride)) binder by a simple wet-chemical method. The textiles were washed with pure water and ethanol several times, treated in an ultrasonic bath for 10 minutes to remove surface impurities, and dried in air at 60 °C. PVDF powder (2.5 g) was dissolved in 50 mL of acetone solution and stirred at 60 °C for 1 h. T-ZnO nanowires (5 g) were added in the PVDF gel and stirred at 60 °C for 1 h. Finally, a uniform T-ZnO/PVDF paste was loaded on the pre-washed textiles (15 × 10 cm) and dried at 60 °C in air. Moreover, the textile size can be cut arbitrarily according to the actual application.

### 2.2. Fabrication of a Self-Powered Device

Two copper foils were bonded to T-ZnO nanowire textiles at different sides as two electrodes, and the device was simply fabricated. A pre-cleaned Kapton board was selected as the flexible substrate for supporting the device in the test. The T-ZnO nanowires were then modified with LOx (supplied by Sigma Chemical Co. St. Louis, MO, USA). 0.5 mL (10 g/L) LOx solution was slowly dropped onto the surface of T-ZnO nanowires and dried naturally (~2 h). This enzyme modification procedure was repeated for 4 times to firmly attach the LOx.

### 2.3. Characterization and Measurement

Scanning electron microscopy (SEM, Hitachi S4800) was used to study the morphology and structure of the nanowires and device. A low-noise preamplifier (Model SR560, Stanford Research Systems) was used to measure the piezoelectric output. The device was first investigated using a programmed stepper motor as the driving force. This device was then attached on the athlete’s body for practical application. All the experimental measurements were conducted at ~20% relative humidity and room temperature (~25 °C)

## 3. Results and Discussion

**Figure 1:**
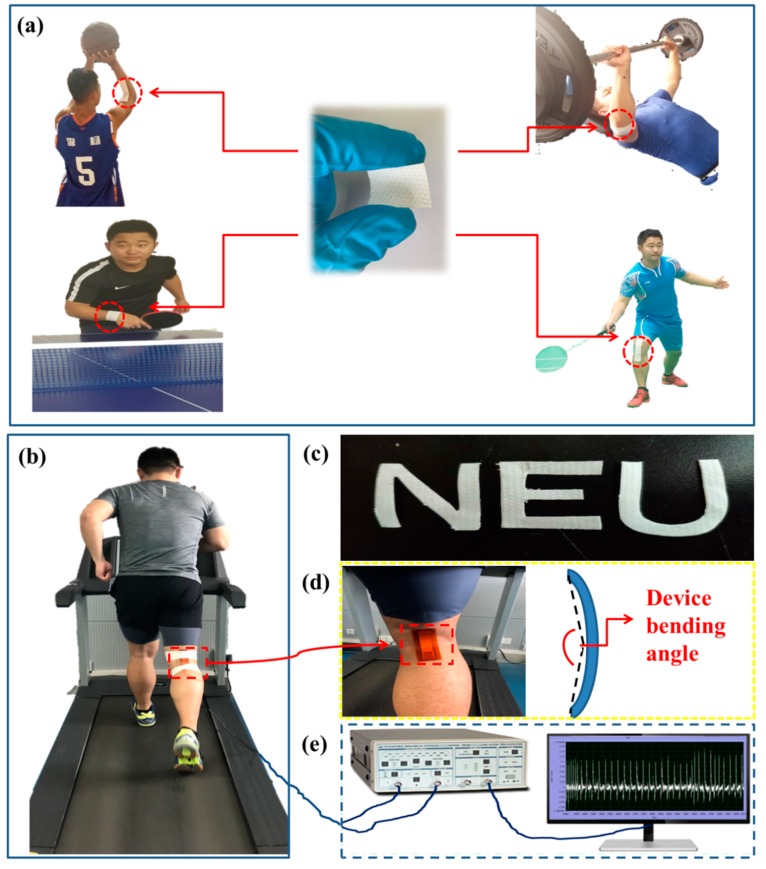
The experimental design of the self-powered piezoelectric-biosensing textiles. (**a**) The practical application. (**b**–**e**) Optical images and the experimental measurement setup.

The experimental design of the self-powered piezoelectric-biosensing textiles is shown in Figure 1. Figure 1a shows the practical application of the textiles [36,37]. The device can be designed and applied according to the needs of different sports projects. Figure 1b shows that the device can be easily attached on the skin surface of a runner at different regions, and the physiological and motor states can be monitored by analyzing motion changes (e.g., joint angles) and sweat lactate. Figure 1c shows that the textiles can be easily cut to different shapes for fabricating distinct devices. Figure 1d is an optical image of the device, showing that the device on an athlete’s leg can be applied with bending deformation during running. Figure 1e shows that a low noise preamplifier and a computer for data acquisition can record the piezoelectric output of the device. 

**Figure 2:**
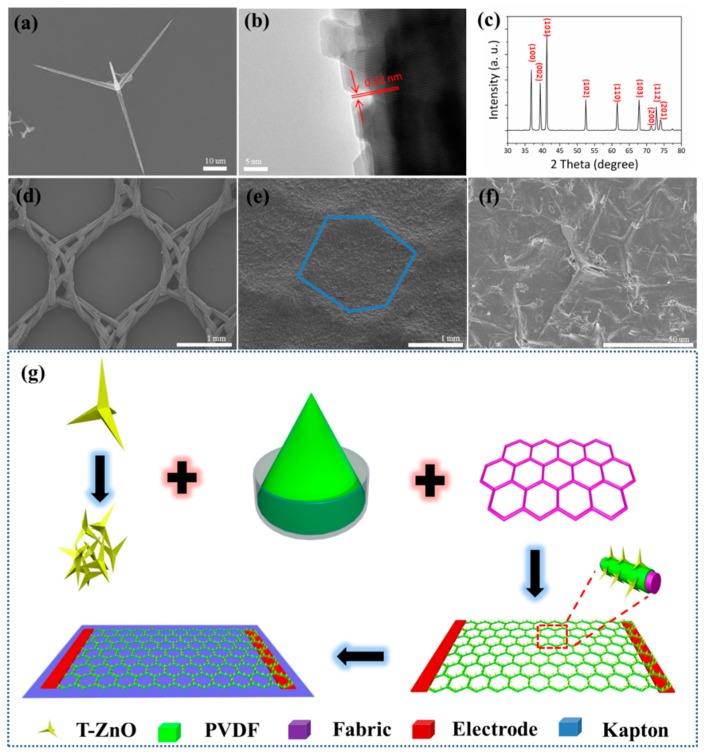
(**a**) SEM image of one single T-ZnO nanostructure. (**b**) High resolution TEM (HRTEM) image of one leg of T-ZnO. (**c**) XRD pattern of T-ZnO. (**d**) SEM image of the textiles (woven fabrics). (**e**) SEM image of T-ZnO/PVDF/fabric. (**f**) SEM image of T-ZnO/PVDF/fabric at high magnification. (**g**) The fabrication process.

An SEM image of one single T-ZnO nanostructure is shown in Figure 2a. It can be seen that T-ZnO nanostructure has a tetrapod shape and a smooth surface. A high resolution TEM (HRTEM) image of the T-ZnO nanowire is shown in Figure 2b. The leg of the T-ZnO grows along the c-axis with a lattice constant of 0.52 nm in the [0001] direction, and the nanowire has a single-crystalline wurtzite structure. Figure 2c shows the XRD pattern of the T-ZnO nanowires, and the sharp diffraction peaks indicate good crystalline quality. The peaks are indexed to the hexagonal wurtzite structure of the ZnO crystal (JCPDS 36-1451), suggesting a high crystal purity. Figure 2d shows a SEM image of the textiles, and the woven fabrics can be observed. The T-ZnO/PVDF can be easily adhered to the fabrics due to the porous structure, as shown in Figure 2e. Figure 2f shows a SEM image of the T-ZnO/PVDF/fabric at high magnification. T-ZnO nanowires are firmly anchored on the fabric, and some legs of the T-ZnO stand out of the PVDF binder, forming a nanoarray structure. The legs of T-ZnO bending with PVDF have strong piezoelectric characteristics and improved biosensing performance [30]. As shown in Supplementary Figure S1c,d, the output voltage and response of the T-ZnO/PVDF/fabric textile is much higher than the output voltage and response of PVDF/fabric textiles. PVDF is just a piezoelectric material, and the surface state cannot be affected by the enzymatic reaction. Thus, the biosensing performance of PVDF is poor. Further, it should be noted that the piezoelectric output of PVDF without polarization is not high, and the pvdf in this paper is mainly used as a binder. Figure 2g schematically illustrates the material system and synthesis process of the device. T-ZnO nanowires are uniformly and firmly attached on the textiles (many woven fabrics). The T-ZnO nanowires with a 3D geometric structure can form a nanoarray on the fabrics. The copper foils on the opposite sides of the device act as the two electrodes. Prior to testing, the device is transferred and bonded evenly to a flexible Kapton as supporting substrate. The detailed fabrication process is discussed in the experimental section.

**Figure 3:**
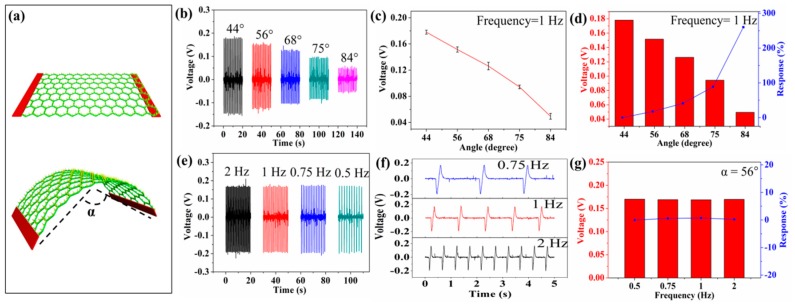
The piezoelectric output of self-powered textiles under applied deformation. (**a**) The bending deformation on the textiles. (**b**) The output piezoelectric voltage of the device at different bending angles (frequency is maintained at 1 Hz). (**c**,**d**) The relationship between piezoelectric voltage and bending angle. (**e**) The output piezoelectric voltage of the device at different frequencies (the bending angle is maintained at 56°). (**f**) The details of the voltage signal. (**g**) The relationship between piezoelectric voltage and frequency.

Figure 3 shows the piezoelectric output of the self-powered textiles under the applied deformation. In this test, the deformation is applied on the device by the stepper motor, as shown in Figure 3a. The two copper electrodes are tightly fixed by clamps and the device mainly bends at the center. Figure S2 shows the piezoelectric voltage of one device after one day and three months, respectively. The operation distance of the stepper motor can be adjusted to control the bending angle of the device. It can be seen from Figure 3b that the output piezoelectric voltage of the device decreases as the bending angle increases (the bending frequency remains at 1 Hz). When the bending angles are 44°, 56°, 68°, 75°, and 84°, the output piezoelectric voltage of the device is 0.178, 0.151, 0.126, 0.094, and 0.049 V, respectively. Figure 3c shows the relationship between piezoelectric voltage and bending angle. The response of the device can be calculated from the following equation:

(1) $$R\%=\left|\frac{V_{0}-V_{i}}{V_{i}}\right|\times 100\%,$$

where V_0_ and V_i_ are the output piezoelectric voltage of 44° and other angles. Figure 3d shows the response of the device is 0, 17.4, 41, 88.9, and 260 at angles of 44°, 56°, 68°, 75°, and 84°, respectively. As the angle increases, the response of the device increases. The piezoelectric output can be treated as the sensing signal for detecting external applied deformation, and this behavior can be used to monitor the motion of an athlete. This technique can be widely applied to exercise training and can monitor the tiny changes in joint angle (small joint angles that video technology cannot capture) or muscle movement under the skin. It will help athletes improve their exercise techniques and enhance their individual performance. For example, by monitoring volleyball swinging movement in detail (the sequence between the basic steps of the action), the volleyball player can improve his or her relevant skills.

Figure 3e shows the output piezoelectric voltage of the device at different deformation frequencies (the bending angle is maintained at 56°). Figure 3f shows the details of the signal. It can be seen that the frequency of the piezoelectric signal is determined by the frequency of the deformation. Thus, the device can be applied to the monitoring of the displacement speed of exercise training. and this technique can be used to point out the influence factors of speed quality, such as the step frequency during running. Increasing the step frequency (the step length is constant) can accelerate the displacement speed of an athlete. Figure 3g shows the response of frequency and piezoelectric output, where V_0_ and V_i_ are the output piezoelectric voltage of 0.5 Hz and other frequencies. When the frequency is 0.5, 0.75, 1, and 2 Hz, the piezoelectric response of the device is 0, 5, 7, and 2, respectively. It can be seen that the values of piezoelectric voltage are substantially constant at different bending frequencies. The output voltage of T-ZnO/PVDF/fabric textiles with and without a Kapton substructure is almost equal, as shown in Figure S1a,b. Thus, the output voltage is mainly attributed to a piezoelectric effect, and the Kapton film is just a supporting substructure. The high and stable output piezoelectric voltage indicates the potential for long-term work.

**Figure 4:**
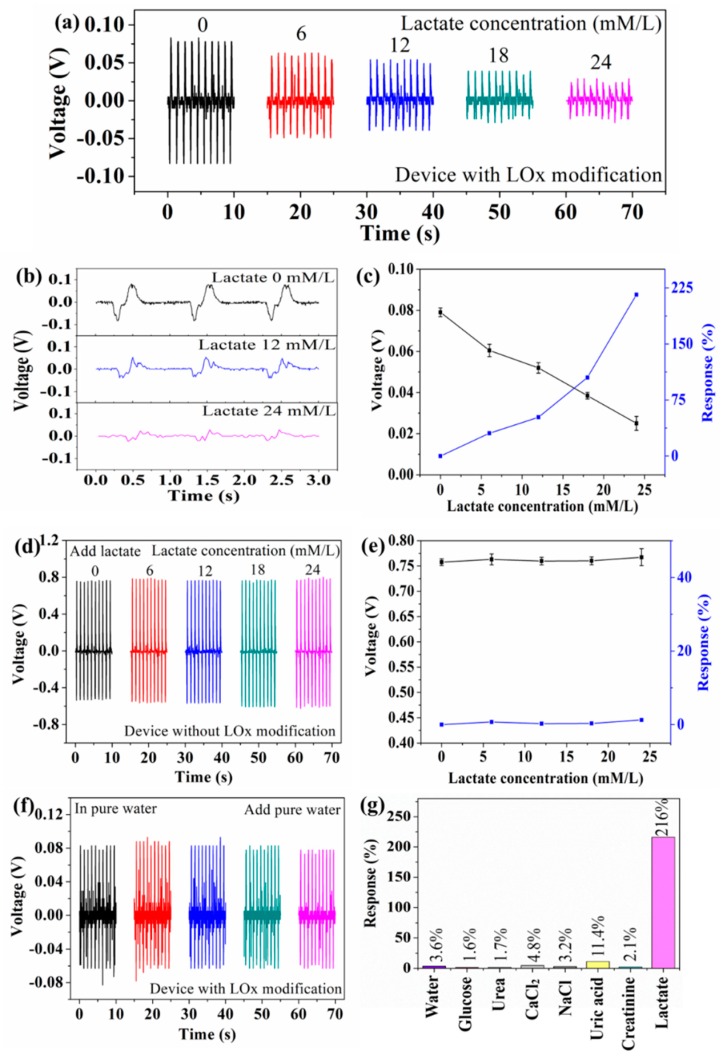
Self-powered piezoelectric-biosensing textiles for detecting lactate concentration in aqueous solution. (**a**) The output piezoelectric voltage of the device (with LOx modification) at different lactate concentrations. (**b**) The details of the signal. (**c**) The response of the device (with LOx modification) against different lactate concentrations. (**d**) The output piezoelectric voltage of the device (without LOx modification) at different lactate concentrations. (**e**) The response of the device (without LOx modification) against different lactate concentrations. (**f**) The output piezoelectric voltage of the device upon adding pure water. (**g**) The selectivity of the self-powered piezoelectric-biosensing textiles.

Figure 4 shows the output piezoelectric voltage of the device at different lactate concentrations (aqueous solution). The device can convert mechanical energy into a piezoelectric voltage signal (serving as both the power and biosensing signal) through the piezoelectric-biosensing coupling effect. Figure 4a shows that the output piezoelectric voltage of the device with LOx modification decreases as lactate concentration increases. As the lactate concentration is 0, 6, 12, 18, and 24 mmol/L, the output piezoelectric voltage is 0.079, 0.060, 0.052, 0.038, and 0.025 V, respectively. The sensitivity limit of the self-powered piezoelectric-biosensing textiles for detecting lactate concentration is 4 mmol/L, as shown in Supplementary Figure S3. Figure 4b shows the details of the signal, indicating that the output value is dependent on the lactate concentration. Figure 4c shows the response of the device to the concentration of 0, 6, 12, 18, and 24 mmol/L. The lactate is 0, 30.5, 51.9, 105.2, and 216.0, respectively (where V_0_ and V_i_ are the output piezoelectric voltage of the device in pure water and lactate solution respectively). A comparison to the previous works is shown in Supplementary Table S1 [25,38,39]. As the lactate concentration increases, the response of the device increases.

The control experimental results are shown in Figure 4d–g, confirming that the biosensing behavior arises from the enzymatic reaction between LOx and the lactate. Figure 4d shows the output piezoelectric voltage of the device (without LOx modification) at different lactate concentrations. As the lactate concentration is 0, 6, 12, 18, and 24 mmol/L, the output piezoelectric voltage is 0.757, 0.763, 0.759, 0.760, and 0.767 V, respectively. Figure 4e shows that the device without Lox modification has no response to the lactate. Figure 4f shows the output piezoelectric voltage of the device upon adding pure water. The output piezoelectric voltage is 0.081, 0.087, 0.084, 0.081, and 0.078 V, respectively. Selectivity is an important parameter for the self-powered piezoelectric-biosensing textiles to distinguish specific biomarkers. Glucose, urea, CaCl2, NaCl, uric acid, and creatinine are the most common components in the sweat, and they are used to test the anti-interference performance of the textiles. As shown in Figure 4g, the response against lactate is much higher than the others (24 mmol/L). These results suggest that the self-powered piezoelectric-biosensing textiles have high selectivity for lactate detection.

Considering that both the bending angle and the lactate concentration influence the output voltage, at the current stage, simply integrating a mechanical device in the self-powered biosensing system can control the bending angle of the textiles for lactate sensing application. For future applications, new device structures and more sensitive/stable material systems need to be designed, so more accurate and reliable lactate detection can be achieved.

In the field of exercise physiology-related training, the lactate parameter has important training significance for assessing an athlete’s aerobic ability and formulating the appropriate intensity of aerobic endurance. By training, the athlete’s lactate threshold (LT) occurrence time can be postponed, and the height of the threshold can be raised. Therefore, the increase of LT is a meaningful indicator for assessing the aerobic capacity of the human body. In addition, endurance training can make breathing and circulatory system functions reach a higher level and also make full use of aerobic energy to minimize the proportion of anaerobic metabolism in energy metabolism. After enhancing an athlete’s aerobic endurance, training intensity should be determined again according to the new LT intensity. Our self-powered piezoelectric-biosensing textiles can measure the lactate concentration in sweat in real-time and facilitate collecting LT data.

**Figure 5:**
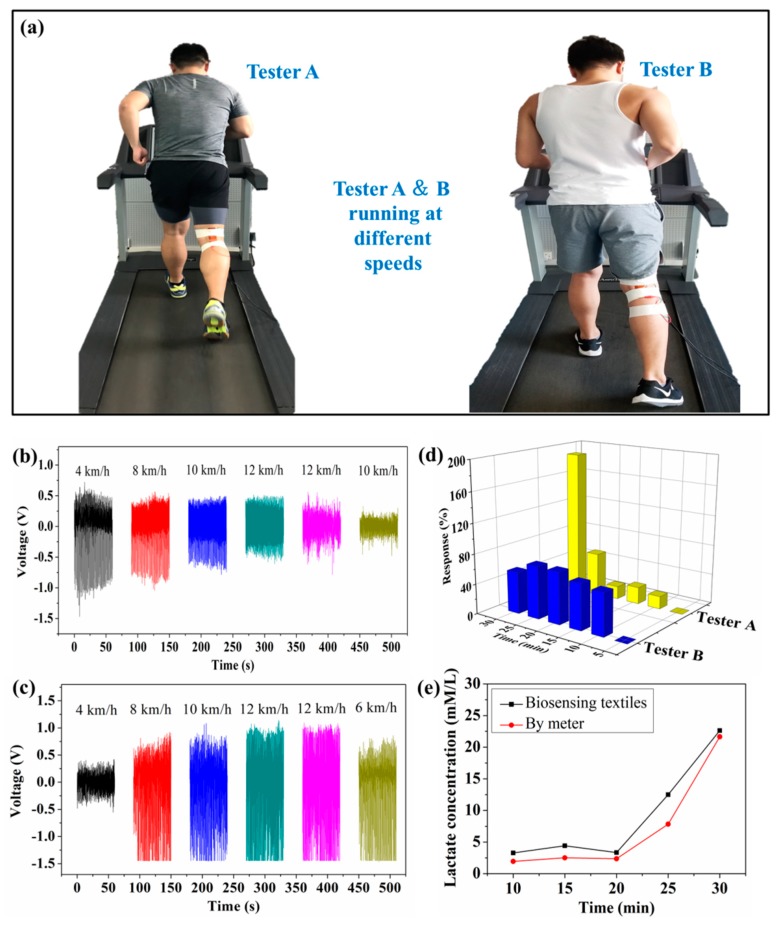
Self-powered piezoelectric-biosensing textiles for monitoring running speed and the physiological state of individual athletes. (**a**) The textiles attached to the two athletes (on the treadmill) for testing. (**b**–**d**) Real-time monitoring testers A and B. (**e**) The lactate concentration measured by the self-powered piezoelectric-biosensing textiles and commercial meter, respectively.

Figure 5 shows that self-powered piezoelectric-biosensing textiles can monitor the running speed and physiological state of individual athletes in real-time. Figure 5a shows the textiles attached to two athletes (on the treadmill) for testing. Both the testers ran for 30 min on the treadmill, and thus the stride length of each athlete was almost kept constant during the test (running speed is mainly dependent on the stride frequency). The leg movement extent of each tester almost kept constant, and the deformation of the device on each tester almost kept constant. The schedule of tester A is 4 km/h (5 min)–8 km/h (5 min)–10 km/h (5 min)–12 km/h (5 min)–12 km/h (5 min)–10 km/h (5 min). The schedule of tester B is 4 km/h (5 min)–8 km/h (5 min)–10 km/h (5 min)–12 km/h (5 min)–12 km/h (5 min)–6 km/h (5 min). Figure 5b shows the output piezoelectric voltage of the device attached to tester A, and the voltage value varies with different running stages. Figure 5c shows the measurement results of tester B, which are obviously different from those of tester A. Figure 5d shows the piezoelectric response of testers A and B throughout the monitoring process. The piezoelectric output is dependent on the sweat lactate concentration and the joint deformation (bending angle and frequency). Thus, the self-powered piezoelectric-biosensing textiles are objective for individual physiological monitoring and time-motion analyses. Figure 5e shows the concentration measured by the self-powered piezoelectric-biosensing textiles and commercial meters, respectively. The results follow the same tend with increasing lactate concentration.

**Figure 6:**
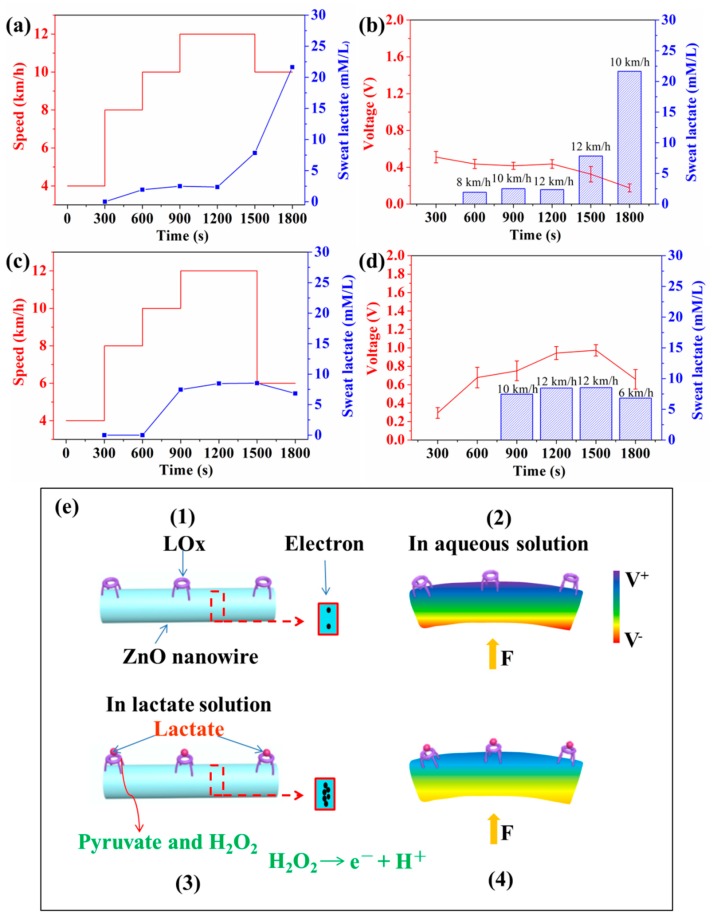
Self-powered piezoelectric-biosensing textiles for sweat-lactate analysis. (**a**) The relationship between the speed and sweat lactate concentration (measured by a commercial meter) for tester A. (**b**) The relationship between speed (lactate concentration) and output piezoelectric voltage of the device on tester A. (**c**,**d**) The experimental data for tester B. (**e**) Working mechanism diagram.

Figure 6 shows that self-powered piezoelectric-biosensing textiles can relatively accurately recognize the sweat-lactate analysis for tester A and B. The tests are conducted on a treadmill. It can be assumed that the stride length of each one almost kept constant during the test, and the deformation of the device on each tester almost kept constant. The self-powered piezoelectric-biosensing textiles are tightly attached to the knee for collecting sports data and the lactate concentration is measured by the commercial meter. Testers A and B are professional and amateur athletes, respectively. Figure 6a shows the relationship between the speed and sweat lactate concentration for tester A. The lactate concentration in sweat is obtained by analyzing the collected sweat with a commercial spectrophotometer. As running speeds of 8, 10, 12, 12, and 10 km/h, the lactate concentration in sweat is 1.94, 2.51, 2.36, 7.84, and 21.64 mmol/L, respectively. It should be noted that during the last 300 s, the running speed of tester A was 10 km/h, and the lactate concentration reached the highest value of 21.64 mmol/L. This stage is not the fastest stage in the whole running process. Tester A had a decrease in aerobic work capacity, and anaerobic metabolism takes place during exercise, enhancing lactate concentration. Thus, the highest LT of tester A is at the end of the quantitative test run [40,41,42]. Figure 6b shows the relationship between speed (lactate concentration) and the output piezoelectric voltage of the device on tester A. In the first 1200 s, the lactate concentration does not change obviously, and the output piezoelectric voltage of the device almost keeps unchanged. As the lactate concentration increases to 7.84 mmol/L (12 km/h) and 21.64 mmol/L (10 km/h), the output piezoelectric voltage of the device decreases to 0.325 and 0.176 V. It can be concluded that the device on tester A can relative accurately determine the sweat-lactate analysis for tester A. 

Figure 6c,d show the experimental data for tester B. As the running speed is 10, 12, 12, and 6 km/h, the lactate concentration is 7.47, 8.46, 8.53, and 6.84 mmol/L. At the speed of 12 km/h, the lactate concentration of tester B reaches the highest value (8.53 mmol/L). As the speed lowers down to 6 km/h, the lactate concentration decreases to 6.84 mmol/L. This phenomenon is very common for amateurs. The highest LT of tester B is at the speed of 12 km/h (1600 s). The anaerobic metabolic energy supply takes place in exercise. Tester B has a low oxygen exercise capacity. The energy produced by aerobic metabolism cannot meet the needs of the body and leads to an anaerobic working state. In the test, tester B showed a physiological decline with decreased cardiorespiratory functions (such as chest tightness and shortness of breath) and could not complete tester A’s plan. During the last 300 s, the running speed had to be lowered to 6 km/h. Figure 6d shows the relationship between speed (lactate concentration) and the output piezoelectric voltage of the device on tester B. The output piezoelectric voltage of the device can relatively accurately reflect the lactate concentration of test B. 

The working mechanism is based on the coupling between the enzymatic reaction and piezoelectric effect (Figure 6e(1–4)). As shown in Figure 6e(1), under the condition that the nanowires are not deformed by external force, the piezoelectric voltage is zero. As shown in Figure 6e(2) (the LOx/ZnO nanowires are not in contact with lactate, and the nanowires are under applied deformation), the enzymatic reaction does not take place on the nanowire’s surface, and the surface carrier density is low. The piezoelectric shielding effect is weak, and the piezoelectric voltage is high. As shown in Figure 6e(3,4), the LOx/ZnO nanowires are immersed in an aqueous solution of lactate, and an enzymatic reaction between LOx and lactate takes place. During the first step, Pyruvate and H_2_O_2_ are produced as follows [43]:

(2) $$Lactate+H_{2}O+O_{2}\overset{LO_{x}}{\to }Pyruvate+H_{2}O_{2}.$$

It has been reported that H_2_O_2_ can transfer electrons into a ZnO nanowire and increase the surface carrier density by producing H+ and e- through the following decomposition process [44]:

(3) $$H_{2}O_{2}\to 2H^{+}+O_{2}+2e^{-}.$$

In this process, H^+^ ion (as extra carriers) adsorption can also take place on the nanowire [45]. Under applied deformation (Figure 6e(4)), the output piezoelectric voltage of the ZnO nanowire is reduced due to the strong piezo-screening effect from the large amount of H^+^ and e^−^ on the surface of the nanowire.

From what has been discussed above, according to the testers’ physiological status, we give the following proposals for reasonable training (Table 1). The late emergence of tester A’s lactic acid threshold, as well as his high LT peak, indicates a strong maximum lactic acid capacity. The recommended training for tester A is interval training to maximize the development of his athletic aerobic capacity, in the case of inadequate rest for the next set of exercises. Tester B’s lactic acid appears earlier, and his LT peak is not obvious, indicating poor aerobic capacity. Continuous training for tester B is recommended to improve his muscle blood supply and oxygen absorption ability, because low intensity training can effectively improve heart function.

## 4. Conclusions

In summary, self-powered piezoelectric-biosensing textiles for individual sports physiological monitoring and time-motion analysis have been developed. Based on the coupling of the enzymatic reaction (LOx and lactate) and piezoelectric effect, the textiles can monitor the moving speed, frequency, joint angle, and sweat lactate concentration of an athlete in real-time. The whole monitoring/analysis process is battery-free. This technique can help different moving objects (individuals) determine their physiological demands and time-motion. This technique can also be used in the scientific selection of excellent athletes and develop appropriate sports training programs for individuals. This work suggests a new research direction for sports science and may stimulate the development of self-powered wearable sports-related systems.

## Figures and Tables

**Table 1:** Testers’ physiological status and training recommendations. LT, lactate threshold.

	Tester A	Tester B
LT appearance time	30 min	25 min
Linear range	0–21.64 mmol/L	0–8.53 mmol/L
Response range	0%–190.5%	0%–69.8%
Training advice	Interval training method: Intensity: Heart rate 170–180/min. Amount: duration <2 min or a few seconds; total amount >30 min. The intermittent time: jogging, bringing the heart rate back to 120/min, and then doing the next exercise.	Continuous training method: Intensity: Low intensity; heart rate 130–145/min. Amount: total amount >30 min. Practice form: running for 2 h, by bicycle or speed skating.

## Supplementary Materials

The following are available online at https://www.mdpi.com/1424-8220/19/15/3310/s1, Figure S1: The output voltages of T-ZnO/PVDF/fabric textiles without/with Kapton substructure, Figure S2: The output voltage of one device after one day and three months, respectively, Figure S3: The sensitivity limit of self-powered piezoelectric-biosensing textiles for detecting lactate concentration, Table S1: The self-powered piezo-biosensing textiles comparison with previous works.
