# Supplementary material for: Self-Powered Piezoelectric-Biosensing Textiles for the Physiological Monitoring and Time-Motion Analysis of Individual Sports
Source: Sensors (Basel). 2019 Jul 27;19(15):3310. doi: 10.3390/s19153310 (PMC6696300; doi:10.3390/s19153310)
Supplement: Supplementary file 1 [file sensors-19-03310-s001.pdf]

Self-Powered Piezoelectric-Biosensing Textiles for the Physiological Monitoring and Time-Motion Analysis of Individual Sports

Yupeng Mao [1], Mailun Shen [1], Bing Liu [3], Lili Xing [2], Song Chen [1,*] and Xinyu Xue [1,2,*]

[1] Physical Education Department, Northeastern University, Shenyang 110819, China
[2] School of Physics, University of Electronic Science and Technology of China, Chengdu 610054, China
[3] School of Arts, Beijing Sport University, Beijing 100084, China
* Correspondence: chensong@pe.neu.edu.cn (S.C.); xuexinyu@mail.neu.edu.cn (X.X.); Tel.: +86-1388-923-0121 (X.X.)

Table S1. The self-powered piezo-biosensing textiles comparison with previous works.

	The power supply mode	The device size	Response range	Detection range	reference
Our work	self-powered	1.5 × 4 cm	0 - 200 +	0 - 25 mmol/L	
Other 1	self-powered	1 × 0.5 cm	0 -95 +	0 - 6.385 mmol/L	25
Other 2	External powered	huge	0 - 5 +	0 - 10 mmol/L	38
Other 3	External powered	2.5 × 7.5 cm	0 - 38 +	0 - 3 mmol/L	39

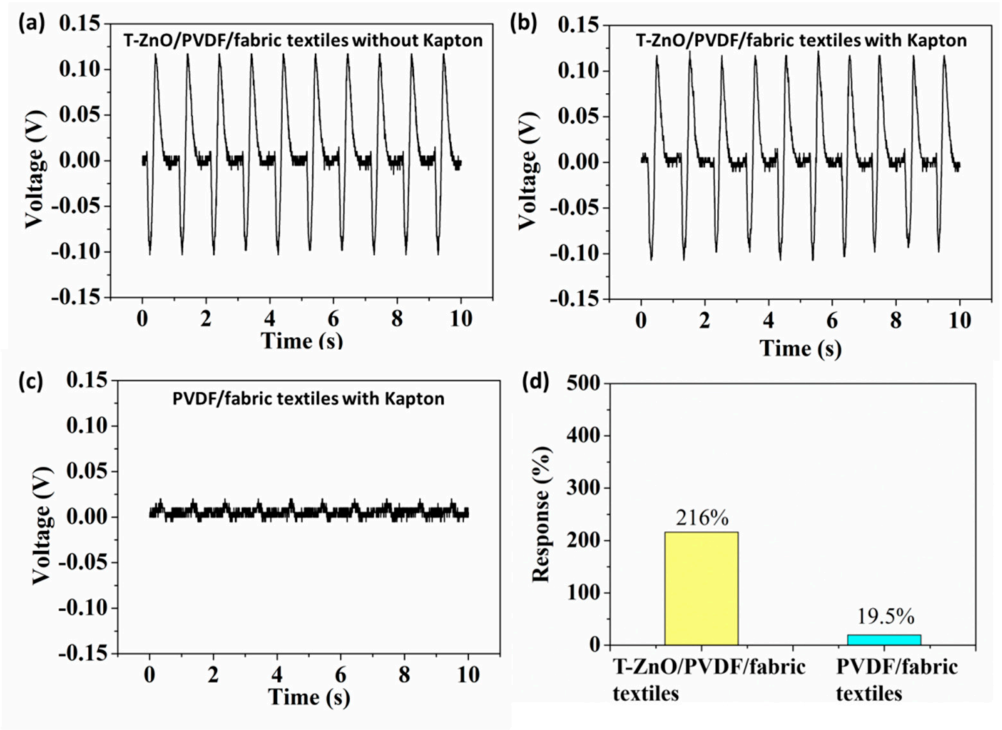

Figure S1. (a) The output voltage of T-ZnO/PVDF/fabric textiles without Kapton substructure. (b) The output voltage of T-ZnO/PVDF/fabric textiles with Kapton substructure. (c) The voltage of PVDF/fabric textiles with Kapton substructure. (d) The response of T-ZnO/PVDF/fabric textiles and PVDF/fabric textiles against 24 mmol/L lactate, respectively.

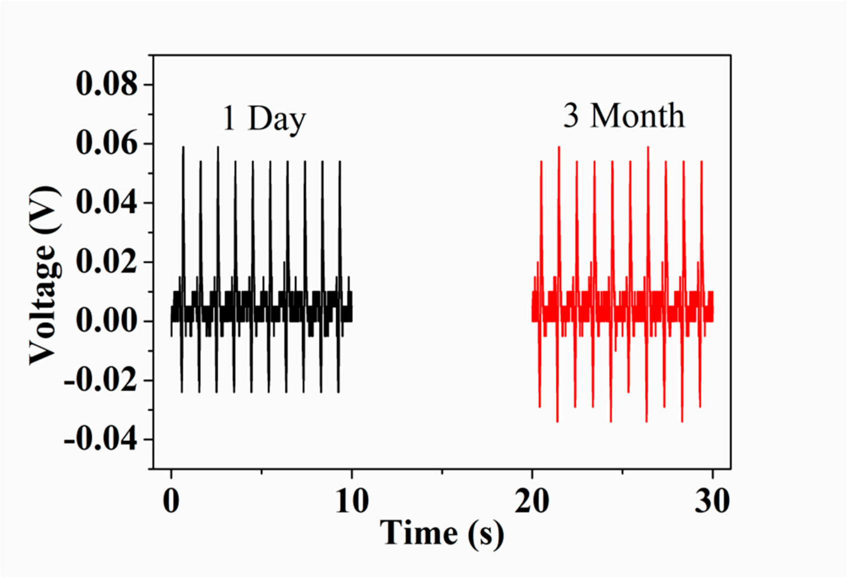

Figure S2. The output voltage of one device after one day and three months, respectively.

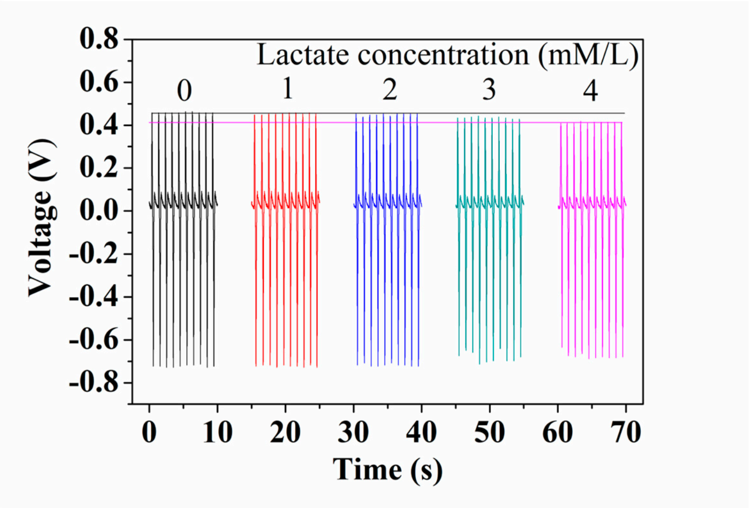

Figure S3. The sensitivity limit of self-powered piezoelectric-biosensing textiles for detecting lactate concentration
